# Health promotion in an Australian Aboriginal community: the Growing Strong Brains^®^ toolkit

**DOI:** 10.1017/S1463423622000020

**Published:** 2022-02-18

**Authors:** Wendy Simpson, Darlene Robinson, Elaine Bennett, Cecily Strange, Vicki Banham, Jenny Allen, Rhonda Marriott

**Affiliations:** 1 School of Arts and Humanities, Edith Cowan University, Western Australia; 2 Ngala, Perth, Western Australia; 3 Ngangk Yira Research Centre for Aboriginal Health and Social Equity, Murdoch University, Perth, Western Australia; 4 School of Population & Global Health, University of Western Australia, Perth, Western Australia

**Keywords:** Aboriginal families, brain development, culturally appropriate, health promotion

## Abstract

**Aim:**

The aim of this paper is to describe the implementation and evaluation of the Growing Strong Brains® (GSB) toolkit in a remote Aboriginal community in Western Australia (WA) over a 2-year period, 2018–2019.

**Background:**

Ngala, a community service organisation in WA, developed the GSB toolkit in 2014, a culturally appropriate and interactive resource to build knowledge of early childhood development within Aboriginal communities. This was in response to evidence that a higher percentage of children in Aboriginal communities were developmentally vulnerable compared to the rest of the population. The GSB toolkit promotes awareness and understanding of early brain development pre-birth and in the early years of a child’s life.

**Methods:**

The project was underpinned by participatory action research (PAR). Reflective PAR review cycles (*n* = 5) monitored local community engagement, navigated challenges and utilised community strengths. Fifty-nine local service providers attended a 2-day formal training. Data were collected by using various methods throughout the project, including feedback following training, focus groups, surveys, one-on-one interviews using yarning techniques and reflective feedback from the Project Lead.

**Findings:**

Establishing local Aboriginal project staff was pivotal to the success of the project. When delivering services for and with Aboriginal people, it is essential that cultural competence, safety and decision-making is carried through from planning to implementation and evaluation, and involves genuine, respectful and authentic relationships. Sufficient time allocation directed towards building relationships with other service providers and local community members needs to be considered and built into future projects.

The Growing Strong Brains^®^ project is embedded within the local community, and anticipated implementation outcomes were achieved. The support of the local people and service providers was beyond expectation, enabling the building of local capacity, and the development of a common understanding of the key messages from the GSB toolkit to allow integration throughout all levels of the community. This project has been important to build on the strategies necessary to introduce, implement and evaluate the GSB toolkit in other remote Aboriginal communities.

## Introduction

A strong future for children begins before birth and is linked to investment in a child’s early years of development. Decades of research has provided strong evidence about how rapidly the brain develops before birth and into early childhood, setting a foundation for learning, health and behaviour throughout life. ‘The early years of life are characterised by significant opportunity, rapid change and accelerated development which is unparalleled by any other subsequent stage of life’ (Baker, 2017, p.3). Plasticity, or the ability for the brain to reorganise and adapt, is greatest in the first years of life and decreases with age, though continues throughout our lives (Phillips & Shonkoff, 2000; Center on the Developing Child, 2016; Baker, 2017).

Human development is impacted by a range of factors such as: parental and family characteristics; the quality of interpersonal relationships; family structure and functioning; the broader community environment and social supports; nutrition; the physical environment; substance use and misuse; stress; and children’s temperament and early-life experiences. Learning and development commences neonatally and is cumulative, with later development building upon earlier development. Environments in which young children spend their time and their experiences affect their neurophysiological, physical and psychological development, and wellbeing (Moore, 2015; Moore, 2018). When families experience health and socio-economic disparities, the risk of long-term effects on the health and wellbeing of children is increased (Coles *et al.*, 2015). Disadvantage experienced in early life is cumulative, and discrepancies between advantage and disadvantage widen throughout life (Moore, 2015).

Ngala, a community service parenting organisation in WA, acknowledged the need to address health inequalities in Aboriginal communities. Australian Aboriginal families have experienced significant disadvantage over a long period of time and across a range of wellbeing measures, often, as an outcome, experiencing higher levels of vulnerability (AIHW, 2011). Enhancing the capacity of Aboriginal parents will further develop stability and positive relationships within the family (Munns *et al.*, 2018).

Ngala, Midwest and Gascoyne (previously Geraldton Regional Community Education Centre) delivered services in remote communities through the Australian Government’s Parental and Community Engagement Program that were culturally safe, respectful and involved Aboriginal families in programme design. Frequently participants and service providers stressed how important it was that families increased their knowledge of early childhood development, including the language of brain development, to provide opportunities to assist and support them in developing their capacity to meet their children’s needs. If parents and caregivers begin with an understanding of how personal experiences, environmental conditions and developmental biology work together in early childhood, they may be able to influence the roots of lifelong physical and mental wellbeing (Center on the Developing Child, 2010). Acquiring new knowledge about early brain development can help parents think critically about what their children need to thrive and empower them to support the developmental and learning needs of their children (Strobel *et al*., 2019).

The health status of Australia’s Aboriginal children is consistently lower than the general population in all five developmental domains; physical, social, emotional, language and communication (AEDC, 2015). Williamson *et al.* (2019) attributed the gap between Aboriginal and non-Aboriginal children in early childhood social and emotional development to the ‘disproportionate burden of socio-economic and early life health disadvantage’ experienced by Aboriginal children and families (p.10).

To reduce the socio-economic and health inequalities between Aboriginal and non-Aboriginal families, Williamson *et al.* (2019) identified the need for culturally appropriate health and social programmes for Aboriginal families from early life. Working in a culturally safe and respectful way will foster empowerment in Aboriginal communities and demonstrate respect for local knowledge to earn the trust of the community (Cameron *et al*., 2016). In addition, it is important to consider the long-term commitment to relationship building and community development in regional Aboriginal communities, and the challenges when working with families whose lives have been impacted by colonisation (Cameron *et al.*, 2016).

Carnarvon was selected as the site for the implementation and evaluation of the GSB project in 2018–2019. Carnarvon is a remote community approximately 900 km north of Perth, Western Australia (WA), with a population estimated at 4,700 in 2020. All areas of Australia are classified under the Australian Standard Geographical Classification (ASGC) to provide a nationally consistent measure of geographic remoteness to allow comparisons and analysis of data across Australia [Australian Bureau of Statistics (ABS), 2016a]. The five geographical classes of remoteness are based on the measure of proximity and access to services (ABS, 2016a), with Carnarvon classed in the second highest category of remoteness. Carnarvon has a high Aboriginal population (18%) compared to the rest of WA (3.1%) (ABS, 2016b) and is classified as an area of high social-economic disadvantage (ABS, 2018). Approximately 40% of children in the community of Carnarvon are from Aboriginal families, and a higher proportion are developmentally vulnerable in one or more developmental domains (31%) than the rest of WA (19%) (AEDC, 2015).

In 2018, Ngala had an established team of 11 Aboriginal staff in Carnarvon delivering services to children, families and community members. All staff had strong connections in the community and the ability to be part of a collegiate group that could develop a supportive community of practice, a key to building professional competencies (Moore, 2015). It was envisaged that service providers across sectors could use their relationships and opportunities for engagement with Aboriginal families to use the GSB toolkit to start conversations about early childhood development, to share their own stories and learn from each other. Carnarvon was therefore identified as an ideal location to enable Ngala to evaluate the implementation of the tool and the associated training of ‘local champions’.

The aim of this paper is to describe the implementation and evaluation of the GSB toolkit in the Aboriginal community in Carnarvon using a participatory action research (PAR) approach. The project sought to empower and increase local community capacity in the use of the GSB toolkit and to grow and enhance the knowledge and understanding of the protective factors for early brain development in the care and wellbeing of young children.

## Methods

In 2014, following consultation with more than 350 Aboriginal families and stakeholders from across regional and metropolitan WA, Ngala developed the GSB toolkit, a resource to build early brain development knowledge and skills across Aboriginal communities. The GSB toolkit promotes awareness and understanding of early brain development science before birth and in the early years of a child’s life, reinforcing learning within a culturally safe context.

The GSB toolkit is a culturally appropriate, interactive series of images and activities that illustrate and describe significant early brain and child development themes. The GSB toolkit is produced as two resources; a flipchart that can be carried and displayed, and a worker’s manual to assist service providers to provide extra information and activities about the messages in the flipchart. Activities include topics to start conversations and activity ideas with pictures explaining how they can be used. Topics covered in the GSB toolkit include messages about brain growth before birth, the importance of good food, play and learning, and keeping culture strong.

Ngala implemented and evaluated the GSB toolkit in the Goldfields region of WA (south-eastern corner of WA) in 2016. The findings from the evaluation of the toolkit in the Goldfields region added to the knowledge and understanding of culturally appropriate ways of working with Aboriginal families and communities, particularly with a focus on the early years and developmental and health outcomes. Furthermore, the evaluation assisted the determination of how training, mentoring and distribution of the GSB toolkit could occur in other communities. The early and ongoing evaluation of early childhood programmes provides important evidence that informs future iterations, checks assumptions about programme elements and allows for the adjustment of programme delivery (Lombardi, 2018).

PAR was used as the overarching theoretical framework for this project. PAR is a well-recognised methodology for health promotion interventions seeking to achieve social change through the active participation of community members in the planning, decision-making and delivery of programmes (Baum *et al.*, 2006; Nastasi & Hitchcock, 2016; Munns *et al.*, 2017). Specifically, a PAR approach involves a partnership with the local community to ensure cultural grounding, acceptance and validity (Baum *et al.*, 2006; Nastasi & Hitchcock, 2016). In partnership, PAR teams consisting of local community members and staff, and cultural and research representatives, undertake iterative reflective cycles of planning, action and review at intervals during the implementation of the programme (Baum *et al*., 2006).

In Australia, PAR has been successful in the implementation of peer-led programmes in Aboriginal communities in remote (Munns & Walker, 2018) and urban areas (Munns *et al*., 2017). Munns *et al.* (2017) outlined the importance of building relationships and trust as foundational elements of working with Aboriginal communities and has identified the sharing of power and flexibility in adapting programmes for local conditions as key factors in an effective PAR programme.

The GSB implementation and evaluation in Carnarvon was designed using a PAR framework based on the evidence (Munns *et al*., 2017; Munns & Walker, 2018) from PAR programmes in Aboriginal communities in WA. This safeguarded the involvement of local community stakeholders in the community-led planning, implementation and evaluation of the GSB toolkit in Carnarvon. Furthermore, this approach ensured a richness of knowledge and understanding of culturally appropriate ways of working with Aboriginal families and communities.

Implementation principles were also applied to ensure quality of implementation. Key concepts included are as follows: consideration of the programme elements and how this would work in the Carnarvon context; what infrastructure supports were essential to contribute to programme quality and sustainability, regular communication, planning and support with the implementation team; translating progress/results of the project regularly with the local stakeholders; continuous follow-up/coaching of service providers following training; and adaption of resources to suit local requirements (Lombardi, 2018; Størksen *et al*., 2021).

Applying PAR principles, a research steering group of Ngala staff and cultural and university representatives with PAR experience was formed. The steering group subsequently recruited a local GSB Project Lead and identified and approached key Aboriginal community members in Carnarvon. The steering group and Project Lead are all authors on this paper. The Project Lead role was critical to the success and sustainability of the project. As a member of the broader Aboriginal team and trained in GSB, the Project Lead was actively engaged in planning, decision-making, service delivery and evaluation and ensured a strong connection with the local community. This enabled the skills and knowledge of local service providers and community members to be built and disseminated throughout the Carnarvon community in a way that was culturally appropriate.

The Project Lead was required to facilitate training with service providers that included representatives from health, education, and community services, and to conduct workshops, discussions, yarning circles and home visits with community members, utilising the GSB toolkit. Providing follow-up with identified local champions and disseminating community stories and findings in formal and informal culturally safe ways was critical to the role. Furthermore, the Project Lead role was developed to collate data, participate in reflective review cycles with the research steering group and participate in other evaluation activities.

Before commencing the service provider training and community sessions, local Aboriginal organisations and community Elders were consulted to garner their support for the project. The training of local service providers in ways to use the GSB toolkit in the community was undertaken to build local capacity and provide opportunities to develop strong partnerships with other service providers and local community groups. To ensure the project was culturally appropriate, a project protocol was made available in text and pictorial formats to ensure access for all members of the community. A communication plan was developed to guide the strategy for the communication of the implementation and evaluation of the project.

Outputs over the 2-year project included five 2-day training workshops for service providers, 9 sessions for parents delivered at the local adult education institution, 21 community sessions, 2 sessions at the local high for senior students, a workshop for Aboriginal fathers and a workshop for an organisation that works within schools with Aboriginal girls. Data were collected by using various methods throughout the project. This included feedback from service providers at the completion of the workshops (*n* = 54), focus groups (*n* = 2) with trained service providers, an online survey for service providers at the completion of the project (*n* = 18), and one-on-one interviews using yarning techniques with service providers and community members (*n* = 6). The Project Lead also provided regular reports that included reflections. These data informed the action research cycle analysis.

Data were analysed by two of the researchers using a thematic analysis method that identifies and reports on common patterns (themes) across the data set, described by Braun and Clarke (2006) and validated by the other researchers. This method of qualitative data analysis enabled the researchers to consider all of the data collected and provide a rich account of the data (Braun & Clarke, 2006).

Attendees at the training included local community engagement officers from the WA Police Force, facilitators at local Aboriginal playgroups, health promotion staff from several community organisations, child health nurses, Aboriginal health officers, community development officers and staff from local schools. The provision of support and mentoring of local service providers was intended to build the sustainability of the project. Table 1 is an example of the workshop outline provided to service providers. The workshops were led by both Aboriginal and non-Aboriginal facilitators and were provided free of charge due to the availability of funding.

**Table 1.** Service provider workshop outline

Workshop components Day 1	Workshop components Day 2
Brain essentials – brain development from conception to 3 years of age.	GSB toolkit – work through each area through discussions and activities.
Yarning sessions – how to implement GSB into the local community and the First 1000 days statements.	Develop personal action plan.
	Evaluation and reflection

The activities in the training workshops were designed to introduce and connect key messages in the toolkit. Each activity was planned to highlight the importance of concrete and visual aids. All activities were flexible and adaptable for change to suit the target audience. More information was on hand for further investigation for participants. The activities are relatable to the areas in the toolkit and foresee its adaptability where possible to align with any service. For example, one activity included the use of string to show participants how strong connections can be formed when repetitive knowledge is given in the early years, therefore strengthening pathways needed for learning and scaffolding information.

Community sessions commenced following the training of local service providers and reflected the needs of the community to build relationships and foster local ‘champions’ to support the utility of the GSB toolkit and its messages. Trained service providers used their new knowledge gained during the 2-day workshops in conversations with community members and families as a part of their everyday work, often during home visits or when community members were attending the service for other reasons (i.e., health visits). Due to the nature of the community sessions, and often opportunistic conversations with community members, the actual number of community members who benefitted from GSB messages is difficult to quantify. The service provider training and community sessions were co-designed with Aboriginal staff, further modified in the context of the community and with input from Aboriginal people in the community as a part of the review cycles. For example, changes were made to the resources provided at the workshops, including a greater focus on yarning using a traditional round yarning mat, and inclusion of more traditional Aboriginal foods provided at the workshops.

An essential part of delivery for both service provider workshops and community sessions is yarning. Yarning is an appropriate and recognised method to garner narrative data through conversations between two or more people sharing and exchanging information in Indigenous communities (Bessarab & Ng’andu, 2010; Leeson *et al*., 2016). Furthermore, yarning is culturally respectful, can be empowering and aligns well with PAR principles (Fredericks *et al.*, 2011). Yarning is necessary to the implementation process as it purposely creates co-ownership and design. It encompasses all elements of a holistic approach by acknowledging the importance of the child through the parent and family. Sharing knowledge and experiences through yarning supports families in a strength-based approach, celebrating and encouraging the successes as a parent and family unit.

**Figure 1.**
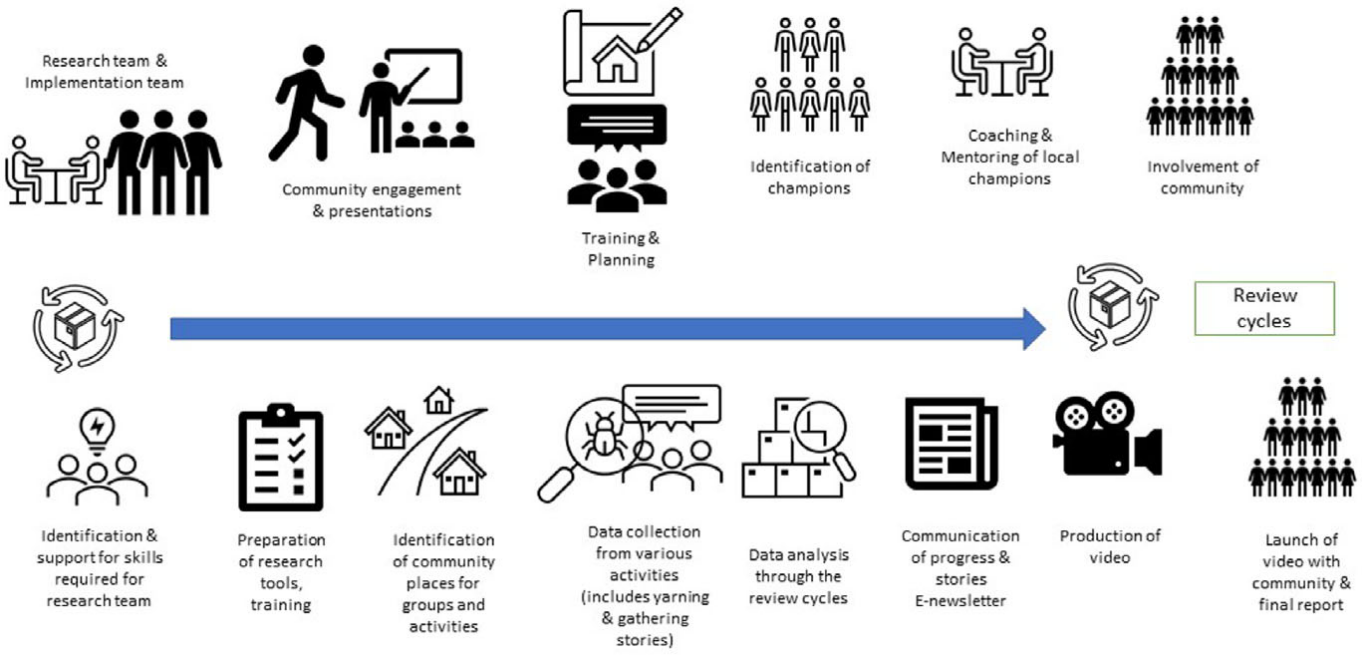
The PAR implementation and evaluation process model for the GSB toolkit

Figure 1 demonstrates the research and evaluation process which engaged community members as collaborators in the implementation and evaluation of the GSB toolkit.

## Findings and discussion

This section summarises and discusses the implementation and evaluation outcomes of the project. The evaluation sought to identify the factors, adaptions and strengths of the GSB toolkit during the implementation in the Carnarvon Aboriginal community to promote skills and knowledge of early brain development in a child’s life. In addition, the evaluation outcomes included evidence to inform future training and mentoring to support the fidelity of the GSB toolkit in other communities.

There was sustained data collection throughout the project. The qualitative data provided rich information from participants’ experiences and opinions about the applicability of the GSB toolkit and messages in their community. Qualitative data collection included online and hard copy surveys, participant observation, yarning sessions and focus groups with service providers who had attended GSB training sessions, and interviews with service providers and community members.

A significant strength of the project was the use of the PAR approach, previously found suitable for Aboriginal communities (Munns *et al.*, 2017; Munns & Walker, 2018). Furthermore, the PAR process and descriptions in this paper provide information that may be applicable and transferrable to other communities. In recent years (Ungar *et al.*, 2015), the role of PAR in implementation science projects has been discussed, and future iterations of GSB implementation in communities may benefit from an integration of PAR and implementation science approaches.

### Consultation and co-design with local stakeholders

The recruitment of a local Aboriginal person to lead the project and the identification and engagement of local stakeholders, Aboriginal Elders, and leaders of the Carnarvon community were essential and commenced early in the project. It was crucial to begin the implementation of the project with full support from the Aboriginal community and the Traditional Group of that area, the Yingarrda people; the language and skin group that is Indigenous to the region. This gave co-ownership and the opportunity to co-design the programme thereby empowering the community to succeed.

### Two-day training of service providers

The training programme for service providers was found to be necessary as a formal introduction to the use of the GSB toolkit and was an essential element to build local capacity to use the GSB resource. The first training session was used as a pilot to refine for further sessions in a local context. The following workshops were tailored to each participant group, but the essential elements of the programme remained for consistency and learning outcomes (see Table 1). A total of 59 individual service providers attended across the 5 workshops, with representatives from health, education and law enforcement sectors, including; the local Aboriginal Medical Service, community police, local government agencies and not-for-profit community organisations, local community health services, early years’ service providers, and staff from local schools. Forty-four per cent of the participants were Aboriginal (26 from 59 participants). Each workshop was facilitated by a Ngala GSB trainer and an Aboriginal Ngala staff member trained in the use of the GSB toolkit. Participants provided feedback about the training sessions and developed their own action plan about how they might use the toolkit in their everyday practice. Participants stated that they gained knowledge about yarning, the impact of smoking, alcohol and poor nutrition on baby’s brain development, and the use of the GSB toolkit as a resource with families. Some participants stated they wanted to use the activities in the workshop with their own families.

Many community members as well as the 59 trained service providers were involved over the life of the project. The versatility of the GSB toolkit meant it can be used as a flexible conversational tool and can be employed throughout a variety of activities from one-on-one conversations, group yarning or organised events. The informal nature of this process did not allow for the collection of the number of community members who gained new knowledge about early brain development and child development from the GSB toolkit. The local service providers built on their knowledge base on the early years and how the key messages can be woven into conversations with community members across the lifespan. Resources for the toolkit were added to as they thought of ideas that can assist to demonstrate these messages with locally based strategies. For example, in a collaborative 6-week programme, the Project Lead and service providers from six local community organisations led a group of Aboriginal women through the GSB toolkit, enhanced by the making of dolls to represent their children, and culminating in a half-day community led ‘cultural tour’ of the Carnarvon region to embed messages of the importance of culture and language. Over time, the team became more confident in the use of the toolkit and learning new ways that were culturally acceptable for working with young women, men and Elders. Following a men’s community group session, one service provider commented:

‘It has made huge differences within the Aboriginal community, particularly the Aboriginal men. For it’s not just the women who have to learn about brain development, also the men’.

### Coaching and mentoring of local champions

The coaching and mentoring of local champions was important to ensure the integrity of the service provider training. Once local champions had undertaken the 2-day training programme, it was important to have regular follow-up and meet with other champions to share their successes and challenges to create a community of practice. This was essential for the sustainability of GSB as ‘everyday practice’.

All service providers who had attended the 2-day training sessions were invited to attend the ‘network meetings’ (focus groups), utilising yarning techniques. Two network meetings were held, facilitated by the Project Lead, with 12–15 service providers in attendance at each meeting. The purpose of these meetings was to provide ongoing mentoring and coaching with their action plans, to gain feedback about the use of the GSB toolkit and to share methods of toolkit utilisation. Examples of new ways to share the GSB messages included card games and books, interactive posters, and cooking sessions with local families and their children. This project found that coaching and mentoring local community members to communicate with families and embed health promotion messages through a cultural lens and in a non-judgemental way was valuable and has been found effective in other studies (Munns & Walker, 2018).

### Action research learning with evaluation cycles

It was anticipated that the evaluation would provide important learnings on how best to foster GSB discussions, increasing understanding of the GSB messages that could support positive behaviour change and optimal early childhood development. A partnership approach to evaluation was taken that involved community members, organisational representatives and researchers. Action learning and evaluation cycles enabled a reflective approach to the project implementation, allowing the participants to ‘take ownership’ of the project (Kelly, 2016). The research team and Project Lead gathered data at each review cycle and discussed what was working well and what needed to change. Each cycle review then had further actions for the following cycle.

The adaptation of the GSB training and toolkit to the local context engendered new ideas and created stories of change that involved the community and reflected their needs. Through the process of training local champions and the delivery of follow-up activities that were generated as a result of the project, stories were exchanged that built on local knowledge and practical ways to translate key messages.

One young Aboriginal man became actively engaged in a workshop practice session, taking on the role of facilitator as part of a scenario with two young pregnant women. He enthusiastically discussed the baby’s growth and development, even before a woman knew she was pregnant. After the workshop, he explained that he had been completely unaware of how much brain development occurred in the first trimester and now understood why it was so important pregnant women did not drink. He initiated a discussion about the importance of partner support and said he would (informally) share these messages with his mates before they became fathers.

In a session delivered at the Shooting Stars, (a local programme that empowers Aboriginal girls and women in regional and remote communities to make informed choices about their education and employment), students participated in an activity to demonstrate the potential effects of alcohol on the developing brain and long-term development. Some students shared stories from a personal space wanting to know more about the possible impacts of drinking alcohol while pregnant, including information about Foetal Alcohol Spectrum Disorder and effects on behaviour.

Sharing food, and stories about food, is common practice for many Aboriginal people: the love of food translates across time and helps establish relationships. During a cultural tour local elders shared stories with service providers about bush foods, including naming foods in the local language. In the workshops, an innovative session of sketching and yarning resulted in lively conversations about food from the past, present and future. These activities provided opportunities to consider how good nutrition supported healthy development and gave service providers a way to translate important messages to their clients about food and nutrition in relation to children’s development that is non-threatening and non-judgemental.

At the end of Day 1 of the workshops, participants were asked to share what they had learned with at least one other person that evening. Several participants returned on Day 2 and said that this had opened up meaningful conversations, for example, with their teenage children, that they had never had before about conception, pregnancy, birth and early childhood development.

### Adapt to local context and follow through new ideas

To foster a collaborative culture, following each of the 2-day training workshops, the facilitators reviewed the process and defined any changes to workshop delivery and content required because of the feedback from workshop participants. It was important to tailor the workshops to the local context to ensure that learning was optimised through community-driven approaches.

Some of these changes included a change of venue to the Gwoonwardu Mia Gascoyne Aboriginal Heritage and Cultural Centre. The GSB Project Lead reflected on the success of this change of venue:

The overall success of the yarning session was the venue. We were given permission to walk through the Cultural Centre during our yarning session. Participants were asked to reflect on early Aboriginal life in the Carnarvon area. Only a few participants had seen the exhibition before. The yarning that happened after the exhibit fully cemented the ideology of cultural awareness training prior to working in a high Aboriginal populace area.

To encourage yarning sessions throughout the training workshop, the introduction of an Aboriginal round mat allowed participants to sit and talk about what they had or hoped to gain from the training. Different strategies were introduced in the delivery of the training workshops, and the lunch was changed from ‘traditional’ to a more culturally contextual lunch for the workshop.

### Find ways to communicate stories of change

Innovative ways to communicate the GSB messages and stories of change were introduced throughout the project. The concept of an e-newsletter for local service providers included information and stories about the use of the GSB toolkit in the community. In each e-newsletter, local community ‘champions’ were identified, and strategies and activities were shared.

During the service provider network meetings, attendees suggested the development of resources, using photos of local families to help them identify with the GSB messages, which led to resources being developed for their own services. During the second year of the project, the concept for a GSB video was developed. The video utilised information from GSB messages with a focus on Carnarvon people and their stories. The video (Ngala, 2019) was launched at a community event and copies were distributed to attendees and throughout the community.

### Strong partnerships with Aboriginal organisations

To ensure the successful implementation of the project, the consultation and development of strong partnerships are essential when working with Aboriginal stakeholders. Throughout the project, partnerships were strengthened between the Project Lead and local Aboriginal organisations. The demand to attend the 2-day training workshops far exceeded expectations. Engaging with community leaders and local Traditional Owners is essential from the beginning and was an ongoing commitment throughout the project implementation. When co-designing community programmes, it is essential that permission is sought from, and given, by local Traditional Owners, using a holistic, community development approach. To develop and build trust with families takes considerable time and a culturally appropriate approach. Recruitment of Aboriginal local people were critical to the success of the GSB implementation. In addition, the support of all local Aboriginal organisations was necessary for the implementation and its success.

### Building on local leadership for sustainability

Capacity building of local leaders within the community contributed to sustainability of the project. Leadership is essential to the sustainability of community programmes. Providing a local Aboriginal person to lead the project, with mentoring and support, has built capacity and passion to continue sharing GSB messages well into the future. Partnerships with local service providers were strengthened because of the training workshops and a ‘community of practice’ was formed. As a sustainability measure, ongoing engagement with local Elders and community members has assisted the enduring utility of the GSB toolkit and its messages.

The programme has been embedded into existing activities, both within Ngala and through external providers. At a regional service provider level, GSB is well known. Monthly meetings of the Midwest Gascoyne District Leadership Group (Carnarvon sub-committee) include consultations around programme design across agencies that deliver services to families, parents, students and community members. For example, a Healing Circle group for mothers is delivered weekly utilising information from the training and the toolkit. Staff from other agencies who co-deliver on specific topics such as family violence reiterate messages from GSB.

The Carnarvon Mental Health & Alcohol and Other Drugs Management Group Plan identified four key priority areas to address local needs. One of the priority areas, *Priority 3*, increase *community knowledge and understanding of Foetal Alcohol Spectrum Disorder (FASD).* Within this plan, prevention and intervention strategies are developed for action in each priority. Local service providers are recruited to implement these actions in relation to their specified service. One of the actions is to deliver and promote GSB.

The Project Lead continues to deliver GSB messages in her role as Project Lead for Every Child, Every Day, a federally funded programme delivered by Ngala in Carnarvon. Programme design is based around GSB activities and then delivered to families, parents, students and community members. The Healthy Bites programme delivered to Pre-Primary children and their families is based around exploring and experiencing new and healthy foods, hygiene and play-based learning.

Service providers across sectors continue to use GSB messages in their daily work. For example, one service provider created her own GSB hygiene booklets as a way to help families understand the importance of daily hygiene and its link to healthy development. Staff at a local employment service, and the staff of a partner Aboriginal organisation, attended the 2-day GSB training to build their knowledge and understanding of the GSB messages to use in their work. Staff now carry the kit with them on home visits to stimulate discussion about parenting and the importance of everyone’s roles in supporting children’s development.

### Identification and management of challenges

One of the benefits of employing a local Aboriginal Project Lead was the acceptance from the local community which enabled relationships to be built and strengthened within the community. However, the time commitment required to continue to engage other service providers and local community members was underestimated in the budget and project plan. This was a challenge for the Project Lead, and strategies were put in place to provide additional support and mentoring throughout the project. Having regular communication and steering group meetings for oversight were important for the ongoing management of issues as they arose. These were documented and reflections formed part of the monthly reports and feedback to the project team. The experiences in this project illustrate the importance of organisational support and responsiveness needed when working with Aboriginal communities found in other studies (Munns *et al.*, 2017).

Flexibility when identifying and managing the challenges of delivering a community-based health promotion programme contributed to the programme success. Challenges facing Aboriginal communities are ongoing and incorporating a process for review and reflection was necessary to assess ‘what was working well’ and ‘what was needing to change’ to ensure that learnings from the project were transferrable to other communities.

### Limitations

The purpose of this project was to implement the GSB toolkit in a remote community, evaluating its suitability and the feasibility of training local champions to deliver key messages around early childhood development, particularly brain development. There was not the capacity, nor the intent, to provide a detailed analysis of outcomes or conduct a comprehensive literature review, but rather to describe the processes and successes so there is the potential to replicate delivery in other communities. The impact of the implementation on outcomes for children and families was beyond the scope of this project and thus was not evaluated.

The focus of the toolkit is on brain development, though does consider this in the context of other aspects of child development and integration with other systems, relationships and attachment, stress, nutrition, hygiene, movement and play, substance use and language and culture. With yarning a significant component of the training, and promoted as the tool for disseminating information, opportunities were provided to discuss other aspects of development such as child and family characteristics, community and social support networks and physical environments. However, it is acknowledged that the toolkit’s messages are framed around brain development, and that recent opinion suggests that a fuller understanding of all biological processes underpinning development may be necessary to avoid lopsided messaging (Moore, 2018). Such considerations were not a part of this project.

Although there is strong anecdotal evidence of sustainability in the use of the toolkit and workshop learnings through ongoing joint service delivery, informal community and service provider feedback and observations, this has not been formally measured.

## Conclusion

This paper has presented the experience, through the Growing Strong Brains® toolkit project, of the application of culturally appropriate and culturally safe approaches of health promotion projects/programmes when working with Aboriginal families in remote communities in WA. The findings from this project were especially significant because of context; for example, remoteness and geographical distance to the research team based in Perth created challenges in regard to face-to-face support for the Project Lead, and the high Aboriginal population and low socio-economic status of Carnarvon.

The outputs from the implementation of the project exceeded those originally anticipated. The learnings suggested that flexibility in approach to consultation and implementation, and the importance of strong partnerships with local Elders and Aboriginal stakeholders, could inform future training and mentoring to support the implementation and fidelity of the GSB toolkit in other communities.

Through the conception, development and implementation, a community development approach focusing on capacity building and authentic relationships with local Elders and local champions, ensured recognition was given to local issues and the impact of socio-economic and health disadvantage in communities. The paper noted the real strength in the Growing Strong Brains® toolkit is in its flexibility when introducing to an Aboriginal community context and in creating strategies to ensure sustainability of the community’s knowledge and understanding of the protective factors for early brain development in the care and wellbeing of young children.
